# Cardiac resynchronization therapy–defibrillator implantation with shock lead placement in the left bundle branch area: a case report

**DOI:** 10.1093/ehjcr/ytae323

**Published:** 2024-07-04

**Authors:** Kenta Yoshida, Mitsuru Yoshino, Tokuma Kawabata, Hiroshi Tasaka, Kazushige Kadota

**Affiliations:** Department of Cardiovascular Medicine, Kurashiki Central Hospital, 1-1-1 Miwa, Kurashiki 710-8602, Japan; Department of Cardiovascular Medicine, Kurashiki Central Hospital, 1-1-1 Miwa, Kurashiki 710-8602, Japan; Department of Cardiovascular Medicine, Kurashiki Central Hospital, 1-1-1 Miwa, Kurashiki 710-8602, Japan; Department of Cardiovascular Medicine, Kurashiki Central Hospital, 1-1-1 Miwa, Kurashiki 710-8602, Japan; Department of Cardiovascular Medicine, Kurashiki Central Hospital, 1-1-1 Miwa, Kurashiki 710-8602, Japan

**Keywords:** Left bundle branch area pacing, Steerable delivery sheath, Shock lead placement area, Left bundle branch–optimized cardiac resynchronization therapy, Case report

## Abstract

**Background:**

Cardiac resynchronization therapy (CRT) with biventricular pacing is a well-established therapy. Left bundle branch area pacing (LBBAP) is a safe technique providing physiological pacing, and LBBAP-optimized CRT (LOT-CRT) has been shown to provide better electrical resynchronization than traditional CRT. However, there are few reports on shock lead placement in the left bundle branch area (LBBA) during CRT–defibrillator (CRT-D) implantation.

**Case summary:**

A 76-year-old woman with heart failure from dilated cardiomyopathy presented with left bundle branch block pattern (QRS duration, 160 ms). Left ventricular ejection fraction was 21%. Cardiac resynchronization therapy–defibrillator implantation was performed due to worsening symptoms. By reshaping the Agilis HisPro catheter and adding a septal curve, the shock lead was placed deep into the ventricular septum, narrowing QRS duration to 114 ms. Left ventricular activation time was 84 ms. A defibrillation threshold test confirmed successful treatment without adverse events. At 6-month follow-up, left ventricular ejection fraction improved from 21 to 63%, with the patient's condition improving from New York Heart Association class III to class I.

**Discussion:**

It was reported that QRS narrowing in CRT was related to long-term mortality, and LOT-CRT further decreased QRS duration as compared with LBBP only or biventricular pacing and increased the response rate. Combining LBBAP with coronary sinus pacing can potentially achieve superior electrical resynchronization. Lack of a suitable tool for direct shock lead placement in LBBA necessitated additional LBBAP lead in conventional LOT-CRT. Our successful LOT-CRT-D procedure with minimal number of leads through Agilis HisPro catheter reshaping enabled direct LBBA shock lead placement.

**Learning points**

- Shock leads from Abbott can be inserted through the Agilis HisPro and can be placed into the left bundle branch area by reshaping them with a second curve towards the septum.
- Achieving LOT-CRT-D is possible, which may further decrease QRS duration compared with LBBP only or BVP and increase the response rate with a minimal number of leads.
- Effective defibrillation is possible even with a shock lead placed in the LBBA.

## Introduction

Biventricular pacing (BVP) is the standard for cardiac resynchronization therapy (CRT) in heart failure (HF) with left bundle branch abnormality (LBBB), as recommended by the guidelines.^1,2^ However, it poses challenges, including a high non-responder rate. Conduction system pacing (CSP)–His bundle pacing (HBP) and left bundle branch area pacing (LBBAP) offer physiological alternatives. Conduction system pacing showed promising results in limited trials.^2^ Furthermore, it has been reported that combining LBBAP with coronary sinus (CS) pacing achieves superior electrical resynchronization [left bundle branch–optimized CRT (LOT-CRT)].^3–5^

While there is a lack of solid evidence regarding whether to implant CRT–pacemaker (CRT-P) or CRT–defibrillator (CRT-D) in elderly patients with non-ischaemic heart disease requiring CRT,^6^ it is necessary to consider the implantation of CRT-D in HF patients with reduced left ventricular ejection fraction (LVEF) who are at high risk of sudden cardiac death, taking into account the patient's prognosis, overall condition, and preferences. Previous reports attempted LOT-CRT using separate pacing leads, complicating implantation.

## Summary figure

**Table:** 

Time	Events
**November 2022**	The patient presented to the hospital with chest pain and difficulty in breathing on exertion. Echocardiography revealed diffuse left ventricular (LV) dysfunction (LVEF: 30%). Electrocardiogram (ECG) revealed the LBBB pattern (QRS duration: 147 ms). Coronary angiography revealed no lesions requiring intervention. Optimal medical therapy was introduced for HF.
**January 2023**	Cardiac magnetic resonance imaging (MRI) was performed; dilated cardiomyopathy was diagnosed. Titration of optimal medical therapy was conducted. She was taking losartan 25 mg, bisoprolol 1.25 mg, and spironolactone 25 mg. The SGLT2 inhibitor was discontinued due to cystitis.
**March 2023**	Electrocardiogram revealed the LBBB pattern (QRS duration: 160 ms). The LVEF was 21.0%. Holter monitoring revealed frequent multi-focal premature ventricular beats and non-sustained ventricular tachycardia. Her low blood pressure made it difficult to uptitrate the medication.
**April 2023**	Cardiac resynchronization therapy–defibrillator implantation was performed.
**June 2023**	At the 1-month follow-up, no adverse events were observed. Multi-point pacing was introduced and optimized.
**October 2023**	At the 6-month follow-up, the LVEF improved from 21 to 63%, and the patient’s condition improved from New York Heart Association (NYHA) class III to NYHA class I. The shock lead parameters were optimal.

## Case summary

**Figure 1:**
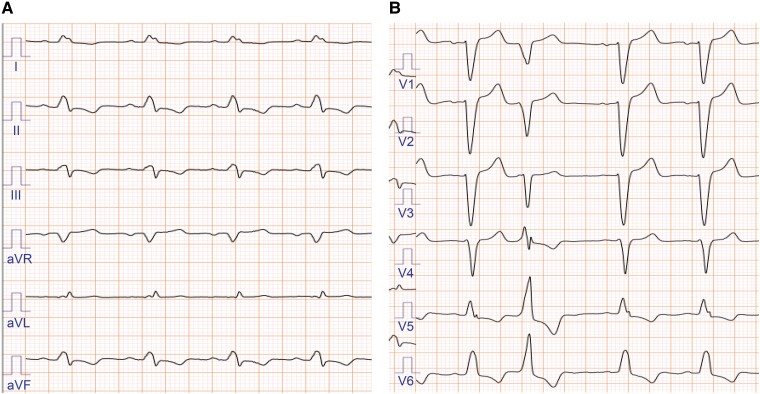
Preoperative 12-lead electrocardiogram. The electrocardiogram showed a sinus rhythm, a complete left bundle branch abnormality, and a QRS width of 160 ms (*A* and *B*).

A 76-year-old Japanese woman with HF due to dilated cardiomyopathy visited our cardiology department, presenting with exertional dyspnoea. Despite optimal medical therapy, she presented with NYHA class III HF. She had comorbidities of hypertension, dyslipidaemia, and diabetes, for which she was taking oral medications and insulin. She had no other past medical history and was independent in her activities of daily living. There were no obvious heart murmurs, and her breath sounds were clear. There was no jugular venous distention, but mild oedema was observed in both lower legs. Coronary angiography revealed no significant stenosis. Her ECG showed sinus rhythm with a LBBB (QRS duration, 160 ms; *Figure 1A* and *B*), and Holter monitoring showed frequent multi-focal ventricular premature beats and non-sustained ventricular tachycardia. Transthoracic electrocardiogram (TTE) revealed a left ventricular end-diastolic (LVDd) diameter of 51 mm, a left ventricular end-systolic (LVDs) diameter of 46 mm, and an EF of 21%. The right ventricular end-diastolic diameter was 29 mm, and the right ventricular fractional area change was 41.9%. There were no significant valvular diseases. Strain rate imaging was not performed. Cardiac MRI showed delayed enhancement in the mid-myocardium of the LV septum, and the extracellular volume fraction was elevated at 31%. Laboratory tests revealed an elevated BNP level of 826.1 pg/mL. Her renal function was preserved with an estimated glomerular filtration rate of 56.1 mL/min/1.73 m^2^, and her serum haemoglobin level was 12.0 g/dL. She was taking losartan 25 mg, bisoprolol 1.25 mg, and spironolactone 25 mg. The SGLT2 inhibitor was discontinued due to cystitis. At the outpatient visit 1 month later, there was no improvement in her HF symptoms, and low blood pressure made it difficult to uptitrate her medications. As a result of shared decision-making, a CRT-D implantation was performed.

**Figure 2:**
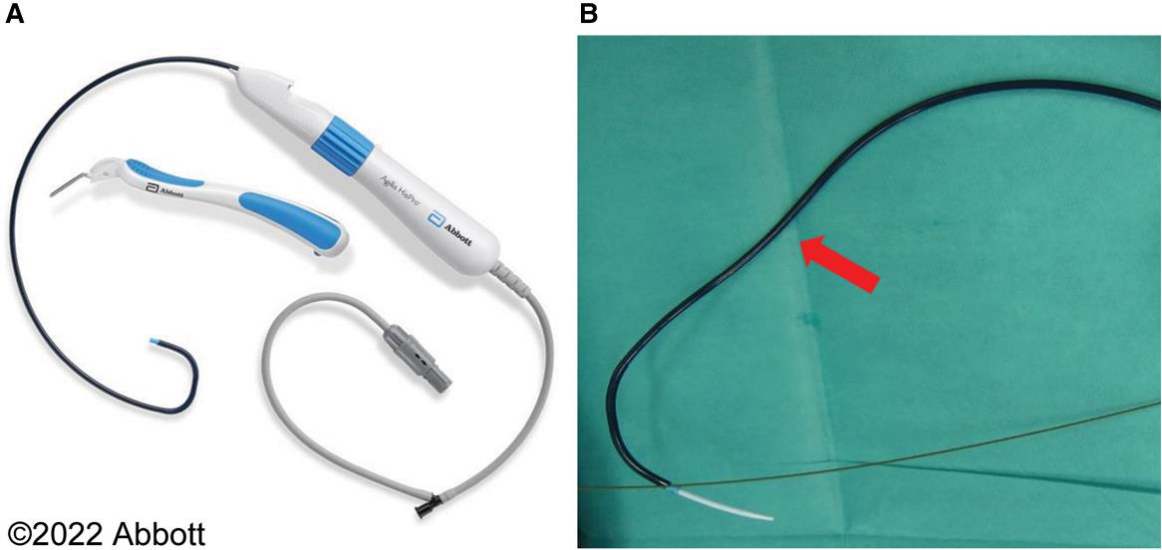
The image shows the Agilis HisPro™ steerable delivery sheath (*A*). The part indicated by the arrow was reshaped in the direction of the septum and made an angle of ∼60° (*B*).

**Figure 3:**
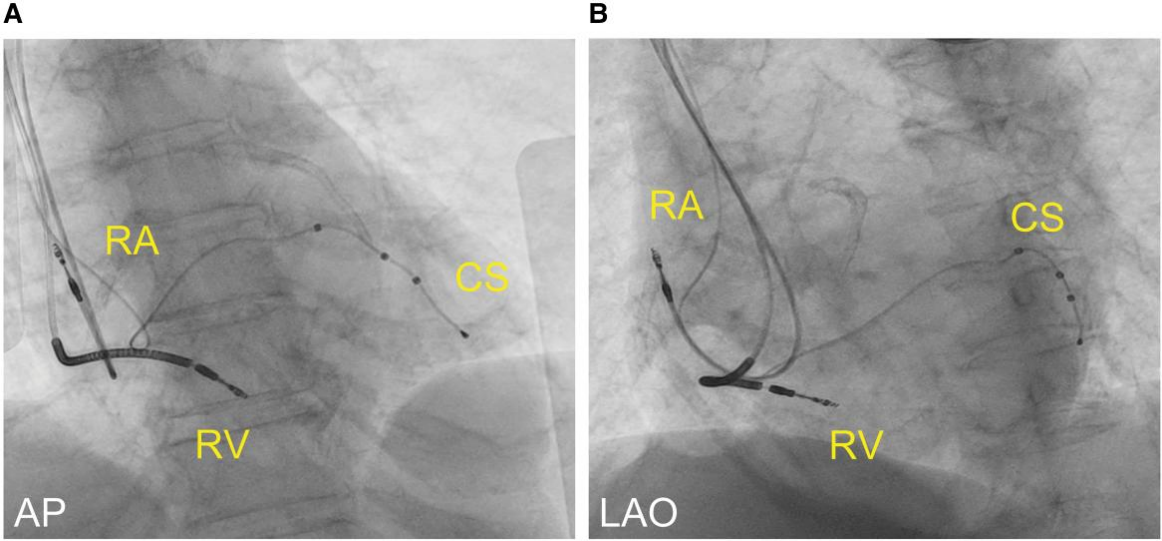
The image shows the lead position after the procedure. The right ventricular lead was screwed deep into the septum using the Agilis HisPro™ (*A* and *B*).

**Figure 4:**
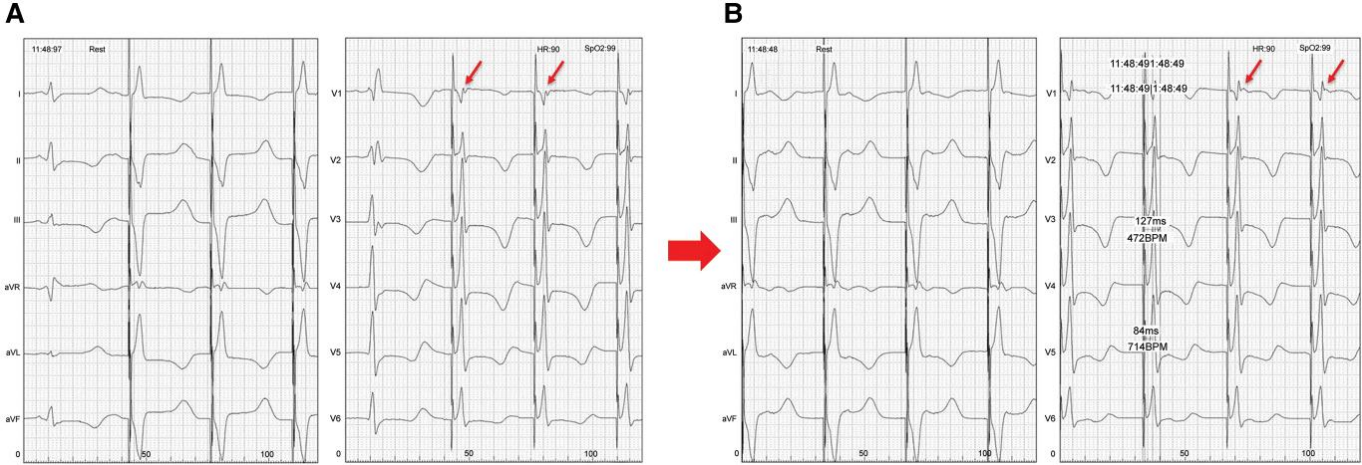
A paced morphology showing a ‘W’ pattern with a notch at the nadir of the QRS in lead V1 (*A*). As the shock lead was gradually screwed deeper into the ventricular septum, the notch in lead V1 was moved from the nadir up to the end of the QRS, and the paced morphology changed to the right bundle branch block (arrows). The left ventricular activation time was shortened (*B*).

**Figure 5:**
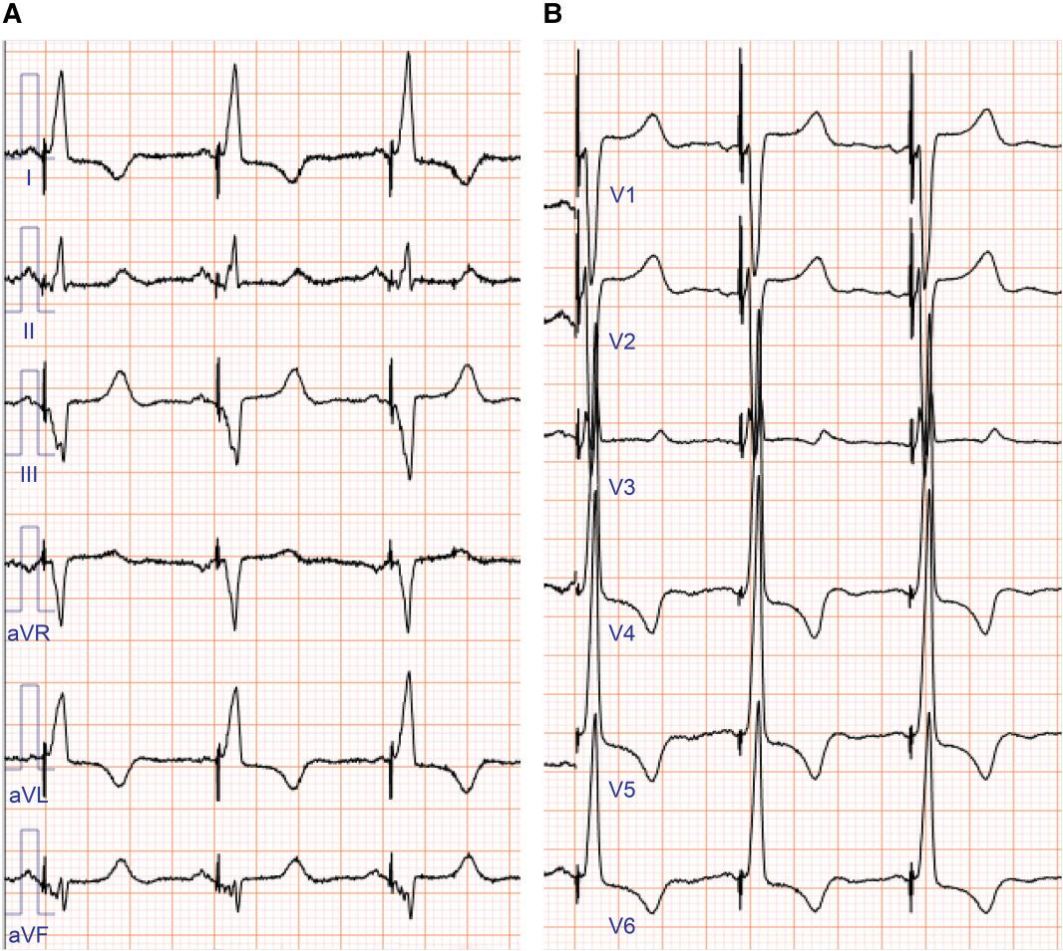
Postoperative 12-lead electrocardiogram. The electrocardiogram showed a QRS width of 112 ms with biventricular pacing (*A* and *B*).

The CRT-D implantation involved placing the atrial lead conventionally in the right atrial appendage and positioning the LV lead in the lateral branch of the CS. We performed LBBAP with a 7 Fr stylet-driven shock lead (DURATA 7122Q, St. Paul, MN, USA) delivered through a steerable catheter (Abbott Agilis HisPro™; *Figure 2A*). Since the Agilis HisPro™ catheter has two 90° deflections, we reshaped its proximal part to the second deflection and added a septal curve (*Figure 2B*: arrow). This reshaping and lead implantation method were performed with reference to the report by Tay *et al*.^7^ The shock lead and sheath were advanced with a slight anticlockwise rotation to guide the shock lead towards the septum (*Figure 3*). The shock lead was advanced with manual rotations applied on the outer lead body. At this point, pace mapping with unipolar pacing at the lead’s tip was performed to assess the presence of a wide ‘W’-shaped QRS morphology in lead V1 of the 12-lead surface ECG (*Figure 4A*). As the shock lead was advanced into the septum, the ‘W’-shaped QRS morphology in lead V1 gradually changed to an incomplete right bundle branch block morphology with continuous monitoring of the unipolar lead impedance and fluoroscopic advancement of the lead into the septum (*Figure 4B*). The shock lead was screwed into the ventricular septum deeply and achieved QRS narrowing of the right ventricular pace (127 ms). The time from stimulus to LV activation was 84 ms. Defibrillation threshold (DFT) test was successful with 15 J defibrillation. The procedural duration was 157 min with fluoroscopy time totalling 53.7 min. No adverse events occurred after the procedure, and the patient was discharged on the 8th day post-procedure. At the 1-month follow-up, multi-point pacing was introduced and optimized, resulting in further narrowing of the QRS width (*Figure 5A* and *B*). At the 6-month follow-up, TTE revealed the LVDd diameter of 40 mm, the LVDs diameter of 32 mm, and the LVEF improved from 21 to 63%. There was no exacerbation of tricuspid regurgitation (TR). The shock lead parameters were as follows: R-wave = 12 mV, impedance = 380 Ω, and threshold = 0.875 V at 0.4 ms. No arrhythmia events occurred, and the ventricular pacing rate was 99%.

## Discussion

This is the first report that LOT-CRT has been established with direct shock lead placement to the LBBA using a reshaped Agilis HisPro™ catheter and QRS narrowing has been obtained. Although the shock lead was placed at the base of the right ventricle (RV) compared to the conventional method, there were no adverse event and the DFT test was passed.

Although there is a lack of solid evidence on whether to implant CRT-P or CRT-D in elderly patients with non-ischaemic heart disease requiring CRT, for HF patients with reduced LVEF who are at high risk of sudden cardiac death, it is necessary to consider implanting CRT-D, taking into account the patient’s prognosis, overall condition, and patient preference. The ESC HF guidelines recommend this with a class IIa indication.^1^ In the present case, despite the expectation of a favourable prognosis due to improved cardiac function, low blood pressure made it difficult to uptitrate medication. Therefore, after shared decision-making, a CRT-D implantation was performed.

Recent reports have shown the efficacy of LOT-CRT.^3–5^ In this method, a separate RV shock lead was still needed to deliver the shock to stop ventricular fibrillation (VF). This finding highlights the potential of using CSP in the CRT-D systems, but the use of more intracardiac leads is required. A combined use of LBBAP and defibrillation of the shock lead could overcome the need for this additional pacing lead, reducing the cost of the procedure and potentially increasing its safety. However, reports on the direct placement of shock leads in the LBBA are lacking. Huybrechts *et al.*^8^ have reported a case series involving the direct placement of shock leads into the LBBA. The success rate of this procedure was ∼60%, and the authors concluded that the manual technique becomes challenging due to the lack of specialized tools.

Therefore, we searched for a suitable tool to place the shock lead directly into the LBBA and discovered that the Abbott shock lead could be inserted into the Agilis HisPro™ catheter. No study has reported the use of this catheter to place a shock lead in the LBBA, but cases of successful RV lead placement to the LBBA by reshaping the Agilis HisPro™ with a second curve towards the septum in pacemaker implantation have been reported.^7^ Using this method, the shock lead was placed in the LBBA with a reshaped Agilis HisPro™ catheter and the procedure was easily performed successfully. This indicates the possibility of establishing LOT-CRT with a high success rate using the Agilis HisPro™ catheter. Patients with reduced LVEF who have indications for both implantable cardioverter defibrillator and pacing should avoid RV pacing and traditionally require placement of CRT devices.^9^ However, notably, the use of sophisticated devices could be avoided using this method.

Limitations of this method include the inability to confirm lead placement using contrast media due to the small difference in lead and sheath diameters.

Performing DFT tests poses potential disadvantages for patients.^10^ Ensuring effective arrhythmia termination with modified lead placement, particularly in the RV base, is crucial. In this study, the shock lead effectively detected and treated induced VF, remaining stable on fluoroscopy with no post-shock parameter changes. However, further case accumulation and data analysis are necessary to confirm the method’s safety.

Furthermore, there were concerns regarding exacerbation of TR due to interference from the shock lead placed at the basal septum. Studies have reported a correlation between the distance from the electrode fixation site to the tricuspid annulus (E–T distance) and lead-related TR, suggesting that placing the lead at a distance of ≥20 mm from the tricuspid valve can prevent exacerbation of TR.^11^ In our case, TTE revealed an E–T distance of 23 mm (see Supplementary material online, *Figure S1*), and no exacerbation of TR was observed at the 6-month follow-up.

Finally, inserting the DURATA lead into the Agilis HisPro and placing it in the LBBA is considered off-label use and requires caution.

In conclusion, we successfully established LOT-CRT-D with a minimal number of leads by directly placing the shock lead into the LBBA with reshaping the Agilis HisPro catheter. However, there were limitations such as not being able to use a contrast agent and requiring DFT.

## Lead author biography


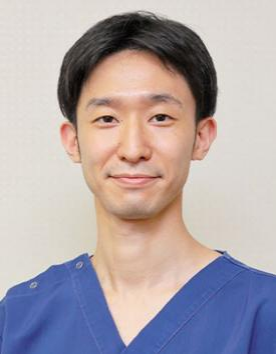


The lead author graduated from Kurume University in 2016 and currently works as a cardiologist at Kurashiki Central Hospital, Japan. His areas of interest include general arrhythmia.

## Supplementary Material

ytae323_Supplementary_Data

## Data Availability

The data underlying this article are available in this article and in its online supplementary material.

## Supplementary material

**Consent:** The authors confirm that written consent for the submission and publication of this case report, including images and associated text, was obtained from the patient in accordance with the COPE guidelines.

**Funding:** None declared.
