# Supplementary figures and images for: Cardiac resynchronization therapy–defibrillator implantation with shock lead placement in the left bundle branch area: a case report
Source: Eur Heart J Case Rep. 2024 Jul 4;8(7):ytae323. doi: 10.1093/ehjcr/ytae323 (PMC11259192; doi:10.1093/ehjcr/ytae323)

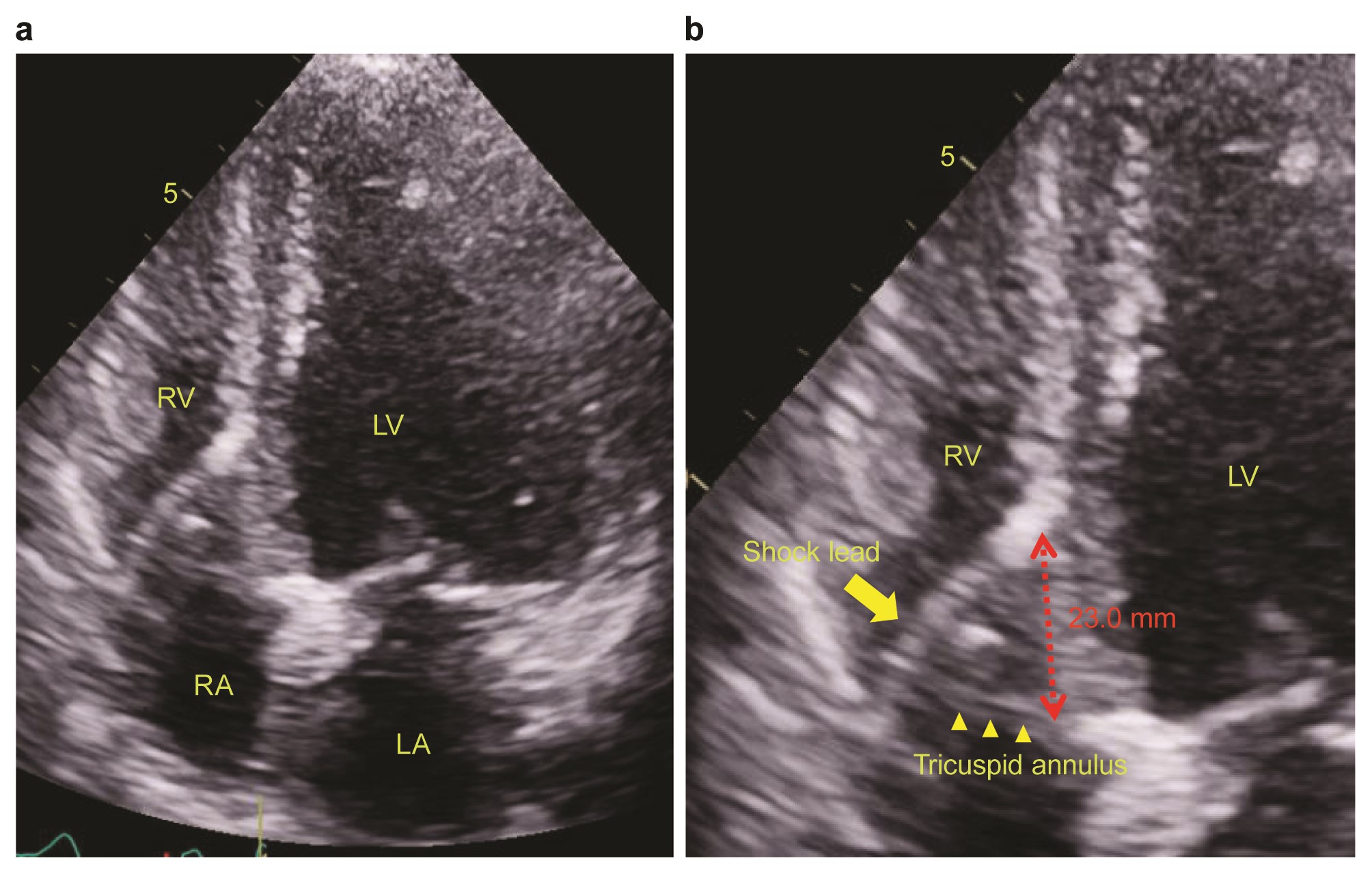

Supplement: ytae323_Supplementary_Data [file ytae323_supplementary_data.jpeg]
